# Activity-dependent neuroprotective protein (ADNP) derived peptide NAP prevents musculoskeletal abnormalities in ovariectomized mice

**DOI:** 10.3389/fnins.2026.1891428

**Published:** 2026-07-28

**Authors:** Liri Sophia Guz, Artur Galushkin, Illana Gozes

**Affiliations:** The Elton Laboratory for Molecular Neuroendocrinology, Department of Human Genetics and Computational Medicine, Gray Faculty of Medical and Health Sciences, Sagol School of Neuroscience and Adams Super Center for Brain Studies, Tel Aviv University, Tel Aviv, Israel

**Keywords:** activity-dependent neuroprotective protein (ADNP), davunetide, estrogen, hormone replacement therapy, muscle strength, NAP, ovariectomy, phytoestrogens

## Abstract

Estrogen is an essential hormone that critically impacts bodily and brain functions, supporting learning, memory, and motor activities. A decrease in estrogen levels is associated with cognitive decline and motor dysfunction, such as muscle weakness. While conventional hormone replacement treatments (HRT) exist, those have limitations and potentially severe side effects. NAP (davunetide) is the smallest neuroprotective peptide site of activity-dependent neuroprotective protein (ADNP), a master regulator of cognition, essential for brain formation. It is known that NAP restores ADNP activity in cases of deficiency and it has already shown potential in preventing cognitive impairment, protecting against tauopathy, and improving motor function in various animal models and in clinical trials. Based on the dynamic regulation of ADNP by the estrous cycle and its involvement in steroidogenic pathways, we hypothesize that NAP may restore ADNP activity and thus serve as an alternative to conventional hormonal treatments. To test this hypothesis, 3-month-old female ICR mice underwent bilateral ovariectomy (OVX) or sham surgery and received daily intranasal administration of NAP, estrogen, or vehicle. Results showed a significant reduction in weight-normalized forelimb grip strength in the OVX model. Interestingly, grip strength was the only test that yielded significant OVX effects, and no significant differences were observed in the novel object recognition (NOR) test or computed tomography (CT) scans. Daily intranasal administration of either NAP or estrogen resulted in an apparent increase in the weight-normalized grip strength compared to the sham-treated OVX group. Furthermore, both the NAP- and the estrogen-treated OVX mouse groups did not statistically differ from the vehicle-treated sham control. These findings suggest that NAP may effectively prevent the loss of physical force production typically seen following ovarian hormone depletion, presenting a viable, non-hormonal candidate strategy for managing musculoskeletal symptoms. We hypothesize that the lack of significance OVX effects in other parameters was due to soy-derived phytoestrogens in the current Tel Aviv University’s standard diet. Thus, the standard diet may have exerted a systemic estrogenic effect that masked the expected physiological phenotypes typically observed in OVX models. Future replication using phytoestrogen-deficient food is required to isolate the specific neuroprotective and musculoskeletal effects of NAP from dietary influence and clarify broader therapeutic benefits.

## 1 Introduction

This manuscript is written for the special Frontiers issue “On the Growth and Form of Animal Behavior—in Memory of Ilan Golani (1939–2024).” On a very personal note, I (Illana Gozes, née Allon) was an undergraduate student at Tel Aviv University during the years 1969–1972, Dr. Ilan Golani was then a young inspiring lecturer in biology teaching us animal behavior, in a memorable course. Years later, as professors at Tel Aviv University, our paths crossed again, and with Ilan together with his long-term collaborator Professor Yoav Benjamini and their former student/postdoctoral fellow, Dr. Neri Kafkafi (Fonio et al., 2012; Kafkafi et al., 2017), together with lead scientists from the Jackson laboratory, we started a collaborative project on a multi-lab experimental assessment. We revealed that replicability can be improved by using empirical estimates of genotype-by-lab interaction (Jaljuli et al., 2023). Our work was partly supported by the US-Israel Binational Science Foundation-US National Science Foundation, BSF-NSF 2016746, and allowed us all to combine animal behavior with strict statistical consideration toward better understanding of biology. Here, in Ilan’s memory we are combining interest in estrogen/sex hormone behavioral and physiological central roles.

Estrogen is an essential hormone that critically impacts bodily and brain functions (Rettberg et al., 2013). It supports learning and memory processes, influences mood, and is important for maintaining bone strength, cardiovascular health, sexual health, and many other functions (Casey, 2017). A decline in this hormone’s levels, whether due to aging during menopause or other physiological conditions, is associated with an increased risk of tauopathy in the brain (Russell et al., 2019) and can lead to cognitive decline, memory problems, decreased motivation, impaired learning abilities, and motor dysfunction, such as muscle weakness or osteoporosis (Cho et al., 2025).

Currently, conventional hormone replacement treatments (HRT) exist, but these treatments have limitations and potentially severe side effects, such as an increased risk of blood clots and cancer development (Tavani and La Vecchia, 1999; Khalifey et al., 2026, Dong et al., 2026). Additionally, there are medical conditions where these treatments are not feasible or recommended. For example, women with a history of breast cancer, especially estrogen-dependent breast cancer, may face an increased risk of cancer recurrence with estrogen therapy. Other examples include men with a history of prostate cancer or individuals of either sex with a history of blood clotting disorders or hypertension, as hormonal treatments may exacerbate these conditions (Tavani and La Vecchia, 1999; Khalifey et al., 2026, Dong et al., 2026). Moreover, some individuals choose to avoid hormonal replacement therapy due to side effects such as bloating, fluid retention, breast tenderness, and reduced fertility in men. While these side effects are not life-threatening, they can negatively affect quality of life and should be considered when initiating treatment (Tavani and La Vecchia, 1999; Khalifey et al., 2026, Dong et al., 2026)

To investigate the mechanisms underlying these estrogen depletion-related declines and to test potential therapeutic alternatives to HRT, researchers often utilize the ovariectomized (OVX) animal model. The surgical procedure bilaterally removes the ovaries, which results in a rapid and significant reduction in endogenous estrogen levels, effectively mimicking the physiological state of menopause in humans (Souza et al., 2018).

In mice, the OVX model reliably reproduces many of the clinical symptoms described in humans. Phenotypically, these mice exhibit a decrease in bone mineral density like human osteoporosis (Xu et al., 2022). This decline is frequently accompanied by a measurable cognitive impairment such as in the novel object recognition test and the Morris water maze test (Tao et al., 2020), and a decrease in the muscle force generation of OVX mice, measured for example in the grip test analysis (Kitajima and Ono, 2016).

Our previous studies identified NAP (NAPVSIPQ single amino acid letter code, also known as davunetide, AL-108, AL-208, CP201 and sometimes referred to as NAP peptide) as the smallest neuroprotective peptide site of activity-dependent neuroprotective protein (ADNP) (Bassan et al., 1999), a master regulator of cognition, essential for brain formation (Galushkin and Gozes, 2025; Pinhasov et al., 2003; Vulih-Shultzman et al., 2007). It is known that NAP restores ADNP activity in cases of deficiency and it has already shown potential in preventing cognitive impairment, protecting against tauopathy, and improving motor function in various models (Karmon et al., 2022; Hacohen-Kleiman et al., 2018), both in animals and humans, such as in studies on progressive supranuclear palsy (Gozes et al., 2023) and pre-Alzheimer’s disease conditions (Gozes et al., 2024; Gozes et al., 2025). NAP and pipeline products protect nerve cells by linking with microtubule end binding proteins (EB1/EB3), through the embedded EB1/EB3 interacting SxIP motif (NAPVSIPQ, Ser-Ile-Pro), thus enhancing microtubule dynamics and Tau-microtubule interaction, protecting the synapse. NAP further enhances ADNP-EB1/EB3, interactions, ameliorating ADNP deficits (Gozes and Shazman, 2023; Hacohen-Kleiman et al., 2018; Ivashko-Pachima and Gozes, 2021).

Importantly, NAP interacts with the neuronal-specific EB1 and not the cancer- associated EB2 (Oz et al., 2014), and unlike estrogen, NAP does not influence cancer cells (Gozes et al., 2003) as further evidenced in extensive toxicology studies and clinical trials (Gozes, 2020; Galushkin and Gozes, 2025). Additionally, with stroke being a side effect of estrogen treatment, NAP was shown to protect in a rodent model of stroke (Leker et al., 2002) and in a clinical trial in patients undergoing coronary artery bypass surgery (CABG), a risk for stroke (Gozes et al., 2025).

While originally characterized by its potent neuroprotective properties and its ability to stabilize microtubules (Ivashko-Pachima and Gozes, 2021), research has identified a significant sexual dimorphism in the expression of ADNP. ADNP expression is not static, rather, it exhibits significant sexual dimorphism and fluctuations in the arcuate nucleus of the mouse hypothalamus with the female estrus cycle. Specifically, ADNP-like immunoreactivity peaks during proestrus, the phase characterized by maximal levels of estradiol necessary to trigger the preovulatory luteinizing hormone (LH) surge (Furman et al., 2004).

Moreover, the importance of ADNP extends beyond its role as a passive marker of hormonal shifts as it appears to be a regulator of the very pathways that produce these hormones. Transcriptomic analyses of ADNP-mutant lymphoblastoid cells have revealed significant alterations in the expression of genes belonging to the steroid-biosynthetic pathway. Gene-set enrichment analysis (GSEA) of these mutants identified “steroid-biosynthesis” as a top hit among significantly over-represented KEGG pathways (*P* < 0.05) (Grigg et al., 2020).

Our recent findings indicated specific Adnp/male regulation of the unfolded protein response (UPR), a cellular stress pathway activated by the accumulation of misfolded proteins within the endoplasmic reticulum (ER) lumen (Shapira et al., 2025). In this respect, both estrogen and testosterone play complex, often opposing roles in the regulation of ER stress and the UPR. Both hormones can either activate or suppress the UPR depending on the tissue, the presence of specific receptors, and the chronicity of stress (Zheng et al., 2016). For instance, estrogen has been shown to protect cells (such as pancreatic beta cells) by repressing apoptotic UPR and enhancing the adaptive UPR activation in response to pancreatic ER stress (De Paoli et al., 2024). Androgenic signaling, such as testosterone and dihydrotestosterone (DHT), drives cellular proliferation and subsequently activates a late-phase, reactive UPR in response to accumulated protein load (Zheng et al., 2016). Importantly, NAP treatment protects against Adnp deficiency (mutation) associated with UPR function (Shapira et al., 2025). Interestingly, this regulation exhibits distinct sexual dimorphism and while UPR dysregulation is found in Adnp mutated male mice, Adnp mutated females display an association with mitochondrial regulation, corrected by NAP treatment (Shapira et al., 2025).

Thus, we hypothesize that NAP (the investigational drug, davunetide) will constitute a novel hormone replacement therapy. Testing our hypothesis in OVX mice indeed showed a significant decrease in grip strength compared to sham treated controls, and significance was lost in OVX animals treated with either NAP or estrogen.

## 2 Materials and methods

### 2.1 Animals

All procedures involving animals have been approved by the Animal Care and Use Committee of Tel Aviv University and the Israeli Ministry of Health. A total of 24 3-month-old female ICR mice were ordered from Harlan and were used in this study. All mice were group-housed, kept on a 12 h light/dark cycle, and had free access to rodent chow and water at the Glasberg Animal Tower of the Gray Faculty of Medical and Health Sciences at Tel Aviv University.

### 2.2 OVX, NAP, and estrogen (17β-estradiol) treatment

Mice were randomly allocated into four experimental groups: Control (sham-operated, receiving vehicle), OVX (vehicle), OVX+ NAP, and OVX+ estrogen. Intranasal treatments were administered in a fixed volume of 5 μL per mouse, 5 days a week, for the duration of the study. The NAP group received 0.5 μg/mouse/dose. The intranasal route was selected to facilitate a non-invasive therapeutic delivery to the central nervous system (CNS) (Galushkin and Gozes, 2025). Intranasal administration was performed by carefully grasping each mouse in a vertical position and applying the solution as un-aerated nasal droplets using a micropipette with a 5 μL tip, following an established protocol (Magen et al., 2014). NAP (davunetide) (98% purity) was obtained from Exonavis Therapeutics Ltd., Tel Aviv, Israel and was dissolved in a vehicle made of sterile water containing 129 mmol/L sodium chloride (7.5 mg/mL), 8 mmol/L citric acid monohydrate (1.7 mg/mL), 17 mmol/L disodium phosphate dihydrate (3.0 mg/mL), and 0.01% benzalkonium chloride (0.2 mg/mL of a 50% solution) (Alcalay et al., 2004). The estrogen group received five daily doses per week of 1 μg/mouse. The estrogenic agent used in this study was 17β-estradiol (>98% purity) purchased from Sigma-Aldrich (St. Louis, MO, United States).

### 2.3 Dietary composition and phytoestrogen analysis

During the experimental period, mice were maintained on the Altromin 1318 diet, which served as the current standard maintenance chow provided by Tel Aviv University’s animal care facility The dietary formulation contains soybean meal, soy husks, and soybean oil. Given that soy-derived isoflavone content can fluctuate significantly based on environmental and harvest conditions, a sample batch-specific analysis was utilized to characterize the phytoestrogen profile of the diet. The analyzed concentrations for the specific representative batch used were 0.362 mg/g Genistein, 0.298 mg/g Daidzein, and 0.068 mg/g Glycitein, resulting in a total isoflavone content of 0.727 mg/g These precise nutritional and chemical parameters were provided directly by the manufacturer (Altromin Spezialfutter GmbH and Co. KG, Im Seelenkamp 20 D-32791 Lage, Germany).

### 2.4 Experimental design and timeline

**FIGURE 1:**
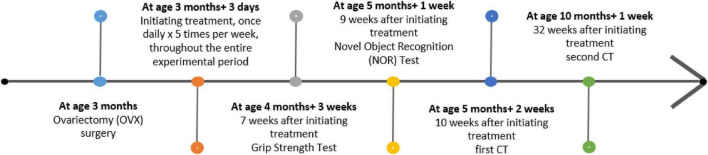
Experimental design and timeline. Female mice (*n* = 0 24) underwent either bilateral ovariectomy (OVX) or sham surgery at 3 months of age. Following a few days of recovery, mice were randomly assigned to four experimental groups: sham+ vehicle, OVX+ vehicle, OVX+ NAP, OVX+ estrogen. Daily intranasal administration was maintained for the whole duration of the study. Functional and behavioral tests including Grip Strength, novel object recognition (NOR) and CT imaging were conducted.

The experimental design and timeline are outlined in Figure 1.

### 2.5 OVX surgery

Surgical ovariectomy was performed under general anesthesia induced by an intraperitoneal injection of a Ketamine (100 mg/kg, Ketamip 10% Veterinary, bela-pharm, Vechta, Germany) and Xylazine (10 mg/kg, Sedaxylan Veterinary, Eurovet Animal Health, Bladel, Netherlands) cocktail. To ensure animal welfare and minimize distress, pre- and post-operative analgesia was administered using Carprofen (5 mg/kg, Rimadyl Cattle, Zoetis, Parsippany, NJ, United States), provided subcutaneously as needed based on previous preclinical assessments (Navarro et al., 2021).

Following the induction of anesthesia, a midline dorsal incision was made, and the ovaries were bilaterally localized, ligated, and excised (Luengo-Mateos et al., 2024). Sham-operated mice underwent the same surgical exposure without ovary removal to serve as a procedural control. All animals were allowed a 5-day recovery period with close monitoring before the initiation of intranasal treatments.

### 2.6 Behavioral tests

All behavioral tests were conducted during the light phase of the circadian cycle in a dedicated, sound-attenuated room. Mice were acclimated to the testing environment for at least 60 minutes prior to the start of each session. All equipment was thoroughly cleaned with a disinfectant solution (Virosol) between trials to eliminate olfactory cues.

#### 2.6.1 Grip strength test

Forelimb grip strength was quantified using a digital Grip Strength Meter [Model 47200, Ugo Basile, Gemonio (VA), Italy]. Mice were held by the tail and lowered over the grip bar, ensuring the torso remained horizontal. Once the mouse firmly grasped the bar with its two forepaws, it was gently and steadily pulled backward until enough tension was created between the mouse and the bar and was held there until the grip was released. The peak pull force was recorded in grams force (gf) by the digital force transducer.

To ensure accuracy and minimize variability, the gauge was reset to zero before each trial. Each animal underwent five consecutive trials in a single session. The three highest recorded values were averaged to determine the final grip strength score for each mouse. This “top three” averaging method was employed to account for fluctuations in mouse engagement or improper attachment during individual trials.

Numerous studies to date have reported that muscle strength positively correlates with body weight, and studies that use muscle strength as an outcome variable usually adopt a method of normalizing this variable by body mass. Additionally, grip strength is correlated with muscle mass. Therefore, absolute grip strength values (gf) were normalized to individual body weight (g), yielding relative grip strength expressed in grams force per gram of body weight (gf/g) (Chun et al., 2019).

#### 2.6.2 Novel object recognition (NOR) test

The test is based on visual discrimination between two different objects in an arena of 50 cm x 50 cm, conducted over three consecutive days. The first day is the habituation day, where each mouse is placed in the empty testing arena for 5 min. This part is also utilized as an open field test. On the second day (the learning phase), each mouse is placed in the same arena with 2 identical objects and allowed to explore them for 5 min, while the time spent sniffing/ touching each object is recorded. On the third and last day (the testing phase), each mouse is placed in the same arena for 5 min each, with one familiar object removed and replaced with a novel object. The time spent exploring each object was recorded and a Discrimination Index (DI) was calculated to determine the preference for the novel object.


$$D\,I=\frac{\begin{array}{c} t\,i\,m\,e\,o\,f\,e\,x\,p\,l\,o\,r\,a\,t\,i\,o\,n\,o\,f\,t\,h\,e\,n\,e\,w\,o\,b\,j\,e\,c\,t-\\ t\,i\,m\,e\,o\,f\,e\,x\,p\,l\,o\,r\,a\,t\,i\,o\,n\,o\,f\,t\,h\,e\,f\,a\,m\,i\,l\,i\,a\,r\,o\,b\,j\,e\,c\,t \end{array}}{\begin{array}{c} t\,i\,m\,e\,o\,f\,e\,x\,p\,l\,o\,r\,a\,t\,i\,o\,n\,o\,f\,t\,h\,e\,n\,e\,w\,o\,b\,j\,e\,c\,t+\\ t\,i\,m\,e\,o\,f\,e\,x\,p\,l\,o\,r\,a\,t\,i\,o\,n\,o\,f\,t\,h\,e\,f\,a\,m\,i\,l\,i\,a\,r\,o\,b\,j\,e\,c\,t \end{array}}$$

A minimum exploration threshold was established- any mouse with a total exploration time of less than 30 s during either the second or the third day was excluded from the final statistical analysis.

#### 2.6.3 Computed tomography (CT) imaging

The mice underwent X-ray computed tomography (CT) using the VECTor4 system (MILabs, Utrecht, Netherlands). Mice were anesthetized with an intraperitoneal injection of a Ketamine (100 mg/kg) and Midazolam (1 mg/kg) cocktail. Quantitative analysis was focused on the femur bone.

### 2.7 Statistical analysis

Statistical analysis was performed using GraphPad Prism version 10.6.1 software.

For the grip strength test analysis, *p*-values were adjusted for multiple comparisons using the False Discovery Rate (FDR) method (Benjamini and Hochberg, 1995) in order to rule out a potential type I error (Kafkafi et al., 2018).

## 3 Results

### 3.1 Grip strength assessment

To evaluate the impact of OVX and the potential protective effects of NAP, forelimb grip strength was measured 7 weeks after initiating treatment, (mice aged 4 months and 3 weeks) and normalized to body weight/mouse.

Table 1 shows the individual body weights, 7 weeks after initiating treatment, which did not statistically differ between the experimental groups.

**TABLE 1:** Animal weights.

F-OVX-NAP (gr/mouse)	F-OVX-estrogen (gr/mouse)	F-OVX (gr/mouse)	F-control (gr/mouse)
29.7	27.6	28.6	29.3
29.7	30.2	29.6	29.7
27.9	32.1	27.4	29.8
30.4	28.9	32.1	31.5
31.1	31.8	26	32
31.4	27.6	28.8	35

Female mice (F) were weighed on the day of grip testing (Figure 1). Individual results are depicted for the four experimental groups.

**FIGURE 2:**
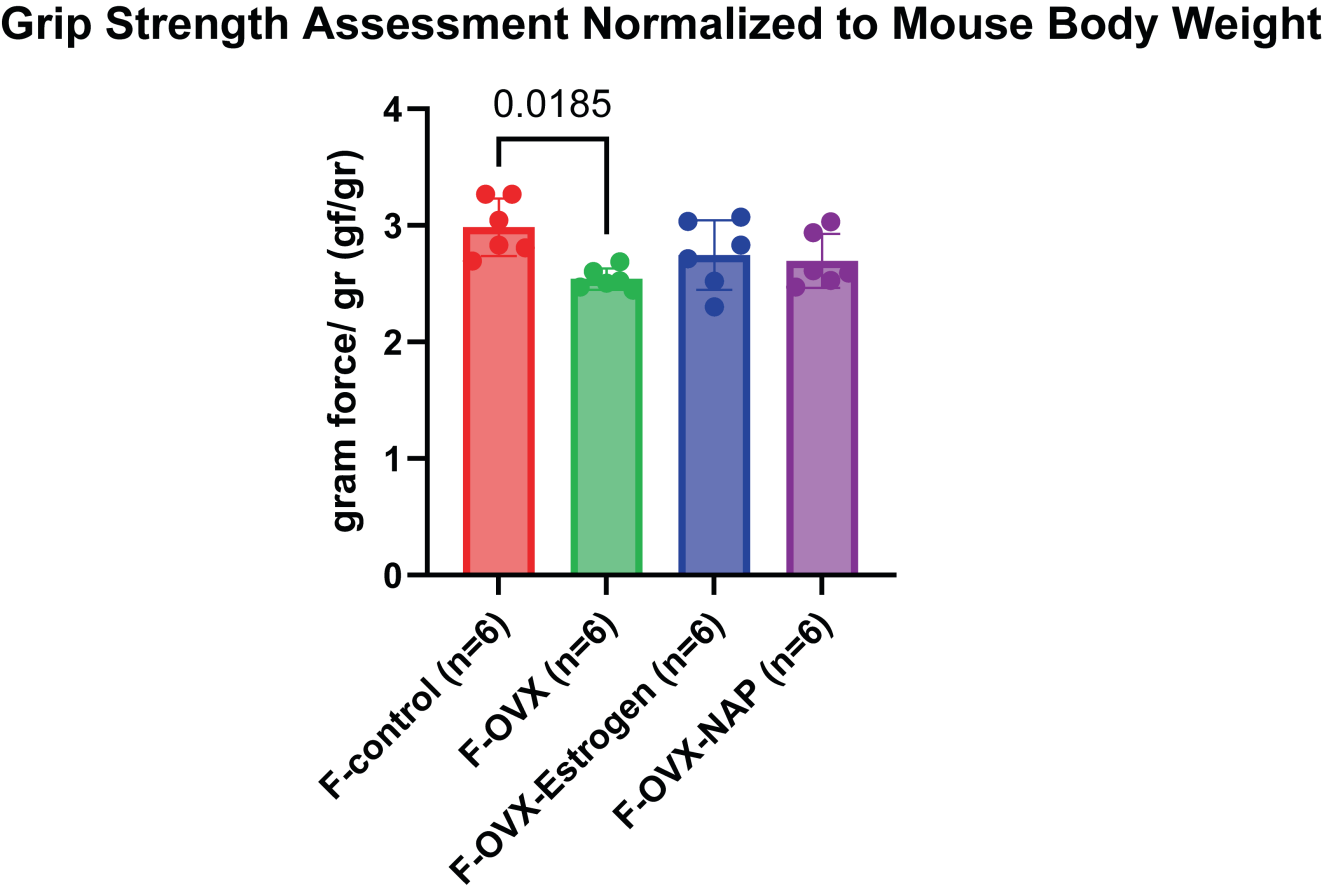
Weight-normalized forelimb grip strength assessment at 7 weeks post-treatment initiation. Forelimb grip strength was measured 7 weeks after initiating treatment and normalized to individual mouse body weight, expressed as grams force per gram of body weight (gf/g, *n* = 6 per group). Data are presented as mean ± SD. Ovariectomy (F-OVX) induced a statistically significant reduction in normalized grip strength compared to the sham-operated control group (F-control, *q* = 0.0185). Daily intranasal administration of either estrogen (F-OVX-Estrogen) or NAP (F-OVX-NAP) resulted in grip strength levels that did not statistically differ from either the control or OVX groups. Data passed the Shapiro-Wilk normality test and differences between groups were analyzed using a one-way ANOVA followed by the original Benjamini and Hochberg method to control the False Discovery Rate (FDR) for multiple comparisons. The desired false discovery rate was set to *Q* = 0.05 (5%).

As shown in Figure 2, OVX induced a minor but statistically significant reduction in forelimb grip strength compared to sham-operated controls (*q* = 0.0185). Daily intranasal administration of either NAP or estrogen resulted in an apparent increase in the weight-normalized grip strength compared to the vehicle-treated OVX group. Furthermore, both the NAP and the estrogen-treated mouse groups did not statistically differ from the sham-vehicle-treated control.

### 3.2 Novel object recognition (NOR) assessment

To evaluate the impact of OVX and the potential protective effects of NAP, the novel object recognition (NOR) test was performed 9 weeks after initiating treatment, when the mice were 5 months and 1 week old.

**FIGURE 3:**
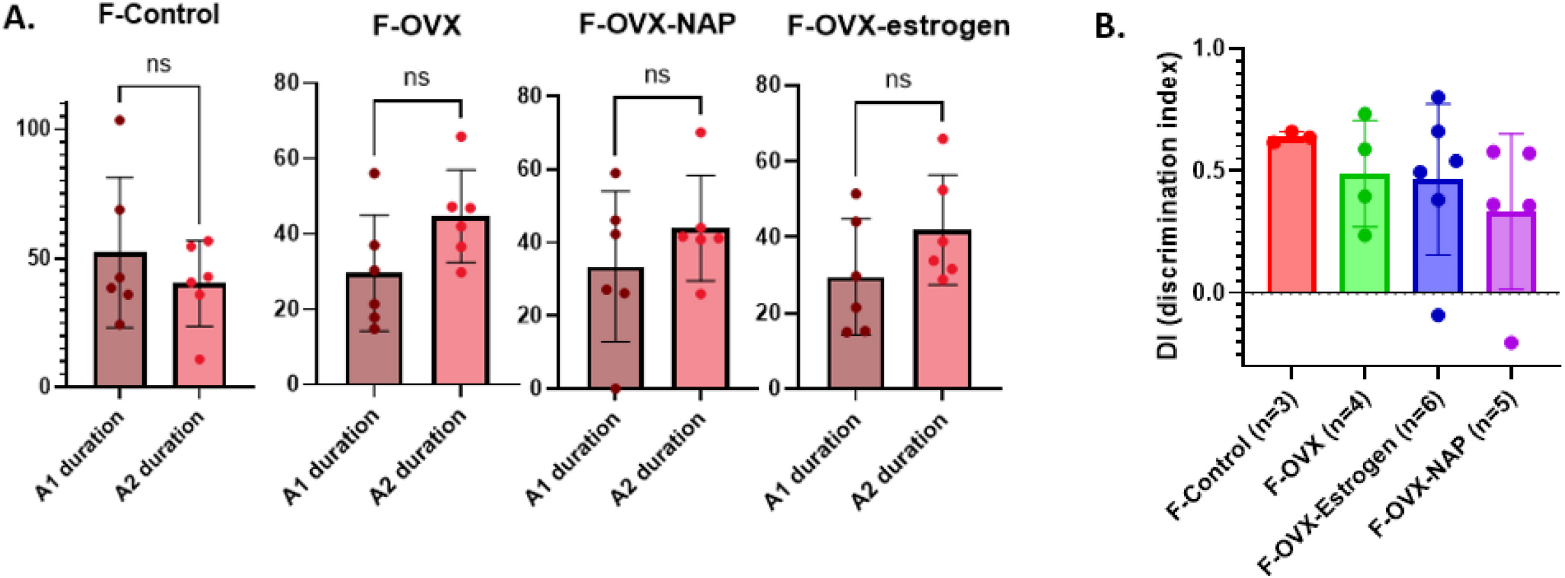
Object recognition assessment at 9 weeks post-treatment initiation. **(A)** Duration (s) spent exploring two identical objects- A1 and A2 during the learning phase was recorded for each mouse. Results show no significant differences between the exploration times for objects A1 and A2 in any group, confirming the lack of innate side bias prior to the testing phase of the NOR test. P calculated by Mann-Whitney U Test. **(B)** Recognition ability was evaluated using the Discrimination Index (DI). The DI was calculated for each experimental group: Control (F-Control, *n* = 3), Ovariectomized (F-OVX, *n* = 4), and OVX treated with NAP (F-OVX-NAP, *n* = 5) or estrogen (F-OVX-Estrogen, *n* = 6). Data are presented as mean ± SD. Results show no significant differences between the groups, indicating that at 9 weeks’ post-treatment, all groups exhibited similar levels of preference for the novel object compared to the familiar object. Data passed the Shapiro-Wilk normality test. Differences between groups were analyzed using a one-way ANOVA followed by the Holm-Sidak *post-hoc* test for multiple comparisons.

On the second day of the NOR assessment, object side preference was evaluated during the learning phase. A two-tailed *t*-test confirmed that there were no significant differences in the duration spent exploring the identical objects A1 vs. A2 across all experimental groups (Figure 3A).

On the third day (the testing phase), recognition memory was evaluated using the Discrimination Index (DI). Statistical analysis showed that all groups exhibited a similar preference for the novel object, and no significant differences were observed between all experimental groups (*P* > 0.05) (Figure 3B).

### 3.3 CT Imaging assessment

**FIGURE 4:**
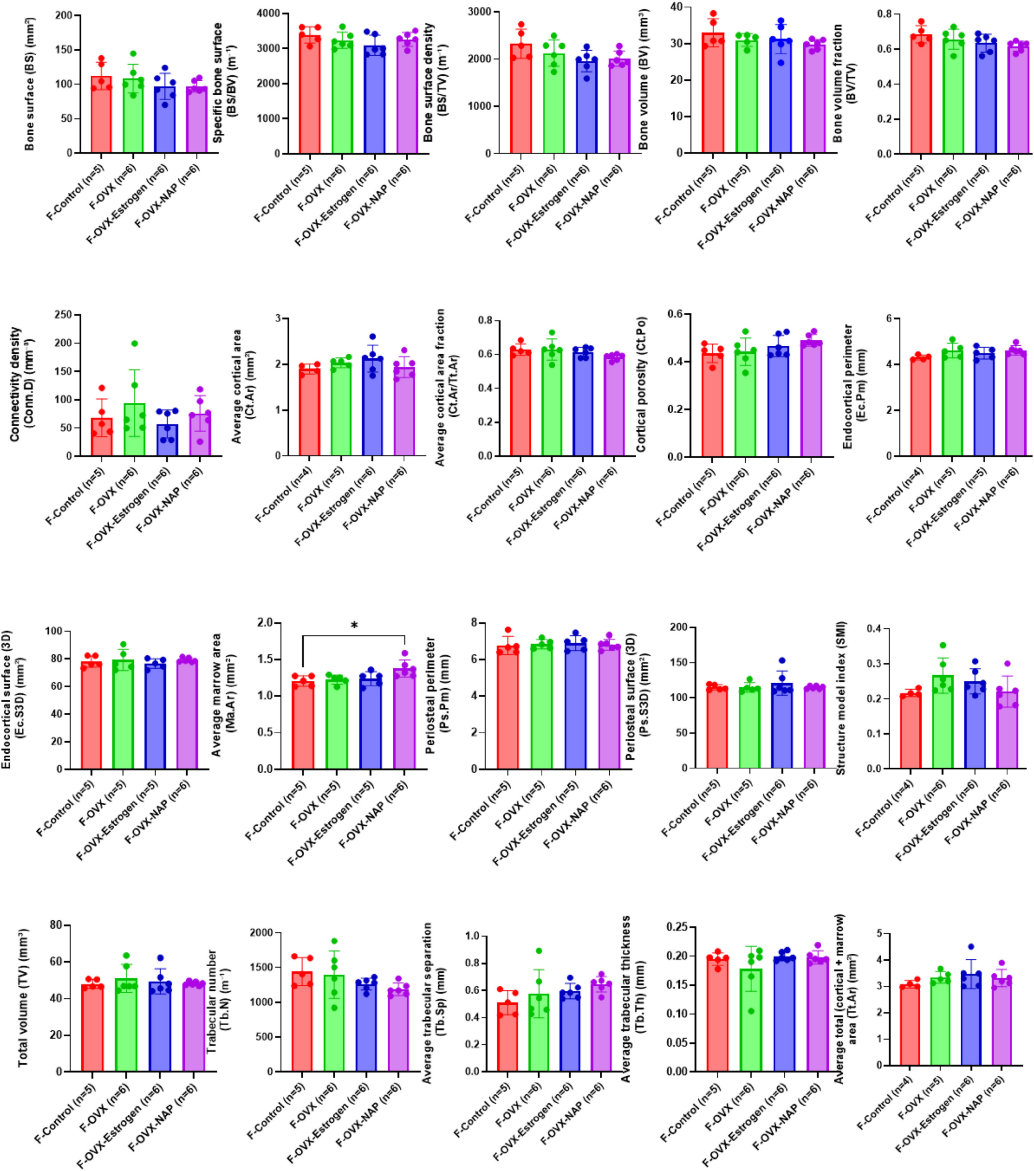
CT imaging assessment 10 weeks post-treatment initiation. Structural bone integrity was assessed 10 weeks after the initiation of treatment. Multiple cortical and trabecular parameters were analyzed for Control (F-Control), Ovariectomized (F-OVX), and OVX treated with NAP (F-OVX-NAP) or estrogen (F-OVX-Estrogen). Data are presented as mean ± SD. Results show no significant differences between any of the experimental groups, indicating that at 10 weeks’ post-treatment initiation, skeletal architecture remained comparable across all conditions. Data passed the Shapiro-Wilk normality test. Differences between groups were analyzed using a one-way ANOVA followed by the Holm-Sidak post-hoc test for multiple comparisons. *P-value < 0.05.

**FIGURE 5:**
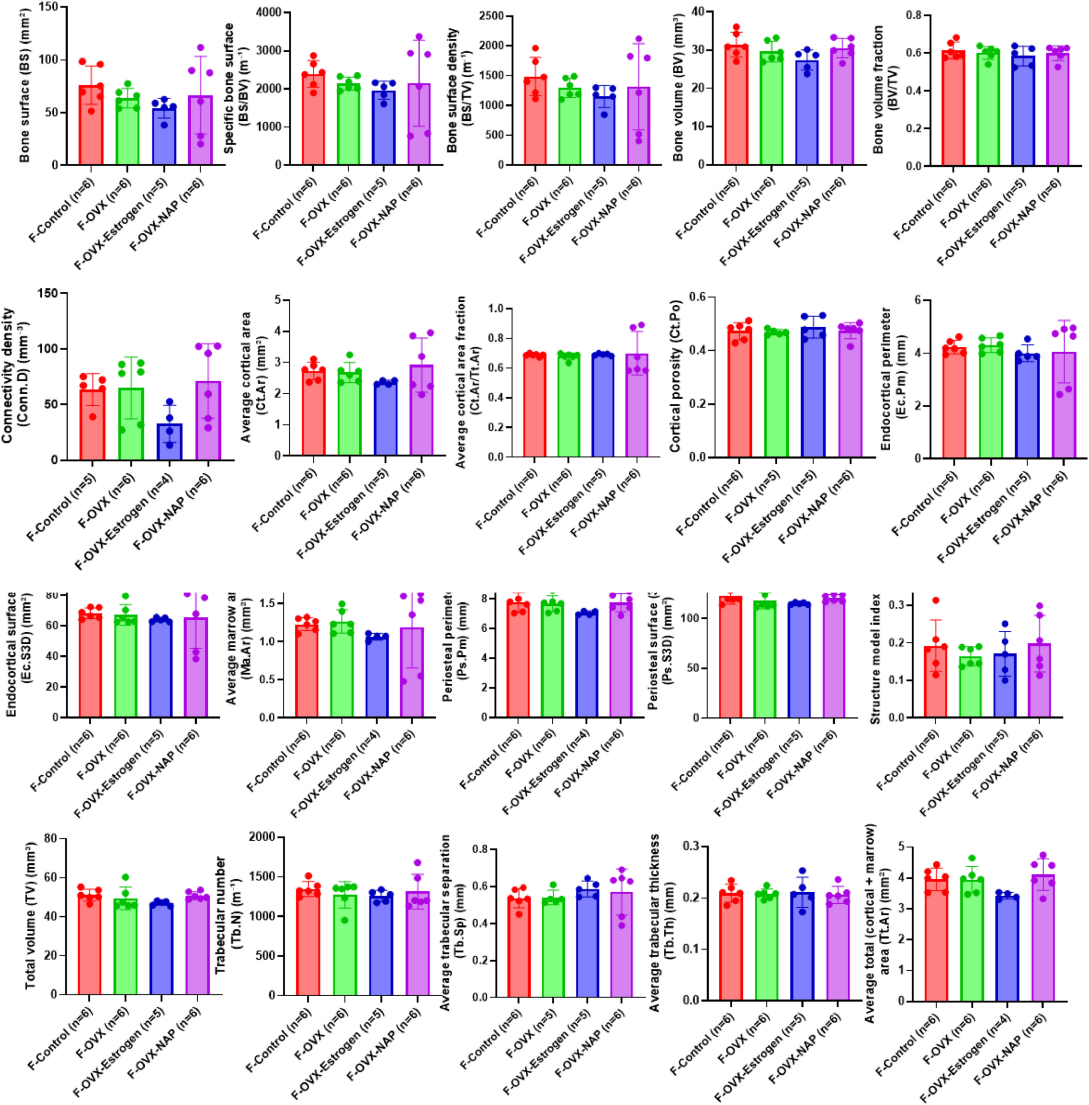
CT imaging assessment 32 weeks post-treatment initiation. Structural bone integrity was assessed 32 weeks after the initiation of treatment. Multiple cortical and trabecular parameters were analyzed for Control (F-Control), Ovariectomized (F-OVX), and OVX treated with NAP (F-OVX-NAP) or estrogen (F-OVX-Estrogen). Data are presented as mean ± SD. Results show no significant differences between any of the experimental groups, indicating that at 32 weeks post-treatment initiation, skeletal architecture remained comparable across all conditions. Data passed the Shapiro-Wilk normality test. Differences between groups were analyzed using a one-way ANOVA followed by the Holm-Sidak *post-hoc* test for multiple comparisons.

To evaluate the impact of OVX and the potential protective effects of NAP, CT analysis of the femur bone was conducted at two different time points including 10 (Figure 4) and 32 (Figure 5) weeks post-treatment initiation.

At 10 weeks post-initiation, no significant differences (*P* > 0.05) were observed between the experimental groups in the evaluated parameters. Moreover, despite the extended duration of the study and the prolonged state of estrogen deficiency in the OVX groups, no significant differences were shown between the experimental groups in the same parameters when re-evaluated at 32 weeks post-initiation (*P* > 0.05).

## 4 Discussion

To accurately assess grip strength, we evaluated force production relative to body weight, directly controlling for individual mass variations. Numerous studies to date have reported that muscle strength positively correlates with body weight, and studies that use muscle strength as an outcome variable usually adopt a method of normalizing this variable by body mass (Chun et al., 2019). Utilizing this mathematical adjustment ensures that the significant reduction in relative grip strength observed in the OVX group (Figure 2) reflects a true decline in functional capacity rather than a passive byproduct of body weight shifts.

This observed deficit in the OVX group likely involves a combination of altered central nervous system (CNS) regulation and intrinsic, structural changes within the musculoskeletal tissue itself.

This hypothesis is supported by recent connectome-based modeling by Ghaffari et al. (2025). In their study, they tested whether they could computationally predict an individual’s handgrip strength, a key clinical biomarker for frailty, by using their fMRI brain scans. Utilizing connectome-based predictive modeling (CPM), they demonstrated that individual variations in physical frailty, specifically handgrip maximum voluntary contraction (MVIC) strength, can be predicted by a person’s unique brain connectome. The study revealed that an individual’s distinct brain-wiring pattern accurately predicts grip strength, identifying the caudate nucleus as the most critical region for predicting physical force. This suggests that “frailty” might start with changes in the brain’s motor networks before it even shows up as major muscle loss.

Furthermore, while NAP is widely documented to enhance synaptic plasticity and stabilize microtubules within the CNS (Karmon et al., 2022; Hacohen-Kleiman et al., 2018), it also exerts direct protective effects within peripheral muscle tissue itself. Single-cell transcriptomics identified ADNP as a major constituent of the developing human muscle, and its systemic deficiency in *Adnp^+/–^* mice has been shown to cause profound neuromuscular junction (NMJ) disruption. Specifically, evaluation of these NMJs revealed significantly decreased microtubule (tubulin) intensities in *Adnp^+/–^* compared to *Adnp^+/+^* mouse muscles, demonstrating a significant genotype-related structural reduction in males, but not in females. Crucially, significant positive correlations were discovered between behavioral test performance and localized *Adnp* gene expression levels in the muscles of 7-month-old mice. Additionally, local muscle deficiency achieved via CRISPR/Cas9 knockdown of adult mouse gastrocnemius Adnp resulted in severe motor dysfunctions that manifested in a distinctly sex-dependent manner, corrected by NAP treatment. Mechanistically, ADNP was found to regulate both muscle microtubules and myosin light chain (Myl2), and these localized deficits were further ameliorated by daily administration of NAP (Kapitansky et al., 2020a,b).

In addition, Adnp acts as a muscle regulator. Quantitative RT-PCR showed that lack of Adnp leads to aberrant gene expression patterns specifically within the gastrocnemius (calf) muscle, disrupting the normal regulation of *Myl* (myosin light chain), a protein essential for muscle structure and contraction. This muscle-specific gene regulation exhibits a significant sexual dichotomy coupled with tight age regulation. Treatment with NAP (davunetide), successfully corrects this altered muscle gene expression, underscoring its therapeutic potential to rescue motor deficits and counteract muscle aging (Kapitansky et al., 2020b). Lastly, human muscle gene-expression database mining demonstrates an association between ADNP transcripts and muscle aging. Specifically, ADNP exhibits increased expression in the *vastus lateralis* of aged individuals compared to young subjects, alongside sex-dependent alterations in the *bicep brachii* of elderly populations, suggesting a direct link to muscle strength and protection against age-associated muscle wasting (Kapitansky and Gozes, 2019).

In the context of our study, the protective trend of NAP then may stem from this dual mechanism of action- its established role in stabilizing microtubules and enhancing synaptic plasticity alongside its stabilization of peripheral muscle architecture.

Interestingly, weight-normalized grip strength was the only test that yielded significant OVX effect in our study. We hypothesize that the lack of significance in the novel object recognition (NOR) and CT scans may be due to the specific composition of the standard rodent diet used in our animal care facility (see Materials and methods, section 3.3). Specifically, this standard diet includes the presence of soy-derived phytoestrogens. These plant-derived compounds, such as genistein and daidzein, bear a striking structural similarity to 17β-estradiol (E2) (Desmawati and Sulastri, 2019), and function as Selective Estrogen Receptor Modulators (SERMs) (Oseni et al., 2008). These compounds may have exerted a systemic estrogenic effect that was sufficient to partially mask the expected physiological phenotypes typically observed in OVX models, thereby narrowing the measurable therapeutic window for NAP.

To address this potential interference and isolate the specific neuroprotective and musculoskeletal effects of NAP from a possible dietary confounding influence, our next experimental step will involve a complete replication of this study design using a phytoestrogen-deficient diet. By eliminating the background estrogenic activity provided by soy-based components, we aim to clarify whether a “cleaner” hormonal environment reveals broader therapeutic benefits across the cognitive and physiological parameters that remained unchanged in the current study.

Finally, it should be taken into consideration that classical estrogen replacement therapy is limited by notable side effects, such as risk of cancer, stroke, and pulmonary thromboembolism (Tavani and La Vecchia, 1999; Khalifey et al., 2026, Dong et al., 2026). In this respect, NAP (davunetide) has already undergone comprehensive toxicological assessments and human efficacy trials, demonstrating a highly favorable safety profile extending twice daily human exposure over a year period, and specific efficacy in neuroprotective, cognitive boosting and motor function conservation in humans, especially in women (Gozes, 2020; Gozes et al., 2023; Gozes et al., 2024; Gozes et al., 2025; Jarskog et al., 2013; Javitt et al., 2012). Further specific to estrogen’s stroke risk (Tavani and La Vecchia, 1999; Khalifey et al., 2026, Dong et al., 2026), NAP was shown to provide neuroprotection in a rodent model of stroke (Leker et al., 2002) and in a clinical trial in patients undergoing CABG, a risk for stroke (Gozes et al., 2025).

## 5 Conclusion

Our data show that NAP (davunetide) might effectively prevent the loss of physical force production typically seen following ovarian hormone depletion. The fact that NAP partially mitigated the decline in normalized grip strength to intermediate levels comparable to estrogen presents it as option for a viable, non-hormonal strategy for managing the musculoskeletal symptoms of menopause.

This is a critical finding for clinical populations where conventional HRT is contraindicated, such as patients with cardiovascular risks or estrogen-dependent oncological histories. Moreover, as for potential treatments in humans, dietary interventions in combination with newly developed intranasal therapeutics, may provide a practical and non-invasive method for managing the physiological deficits caused by estrogen depletion.

## Data Availability

The original contributions presented in the study are included in the article/supplementary material, further inquiries can be directed to the corresponding author.

## In memoriam

This manuscript is dedicated to the memory of Professor Ilan Golani (1939-2024), whose pioneering work profoundly shaped the quantitative analysis of animal behavior. With this paper, we wish to honor his enduring legacy, which continues to influence our laboratory’s approach to studying the intricate growth, form, and expression of animal behavior.
